# Supplementary material for: Synthetic circuits reveal how mechanisms of gene regulatory networks constrain evolution
Source: Mol Syst Biol. 2018 Sep 10;14(9):e8102. doi: 10.15252/msb.20178102 (PMC6129954; doi:10.15252/msb.20178102)
Supplement: Supplementary file 1 — Appendix [file MSB-14-e8102-s001.pdf]

Appendix

Synthetic circuits reveal how mechanisms of gene regulatory networks constrain evolution

Yolanda Schaerli[1,2,*], Alba Jiménez[3], José M. Duarte[2], Ljiljana Mihajlovic[1,2], Julien Renggli[4], Mark Isalan[5,6], James Sharpe[3,7,8], Andreas Wagner[2,9,10,*]

1) Department of Fundamental Microbiology, University of Lausanne, Lausanne, Switzerland

2) Department of Evolutionary Biology and Environmental Studies, University of Zurich, Zurich, Switzerland

3) Systems Biology Program, Centre for Genomic Regulation (CRG), Barcelona, Spain

4) Independent Researcher, St-Sulpice, Switzerland

5) Department of Life Sciences, Imperial College London, London, UK

6) Imperial College Centre for Synthetic Biology, Imperial College London, London, UK

7) Institucio Catalana de Recerca i Estudis Avancats (ICREA), Barcelona, Spain

8) EMBL Barcelona, European Molecular Biology Laboratory, Barcelona, Spain

9) The Swiss Institute of Bioinformatics, Bioinformatics, Lausanne, Switzerland.

10) The Santa Fe Institute, Santa Fe, NM, USA

*Correspondence: yolanda.schaerli@unil.ch; andreas.wagner@ieu.uzh.ch

Table of Content

- **Appendix Figure S1:** Phenotypes measured of all mutants. 3
- **Appendix Figure S2:** Correlation between number of mutations and patterns. 7
- **Appendix Figure S3:** Phenotype diagrams. 8
- **Appendix Figure S4:** Number of mutations for the "red" and "blue" genes. 10
- **Appendix Figure S5:** Summary of all mutations for the "red" and "blue" genes. 11
- **Appendix Figure S6:** An experimental example of how non-additive interactions of mutations in multiple regulatory regions in the concurring gradients network produce a flat phenotype. 12
- **Appendix Model description** 13
- **Appendix Table S1:** Model and fitting of the opposing gradients network 14
- **Appendix Table S2:** Model and fitting of the concurring gradients network 15
- **Appendix Figure S7:** Simulation of spatiotemporal course of gene expression 16
- **Appendix Table S3:** Intervals for parameter mutations. 17
- **Appendix Table S4:** Lengths of the regulatory sequence elements and average mutation rates 18
- **Appendix Table S5:** Data underlying Fig. 6 18
- **Appendix Discussion of lower and upper bounds of the parameter intervals** 19
- **Appendix References** 20

Opposing gradients

"red" gene

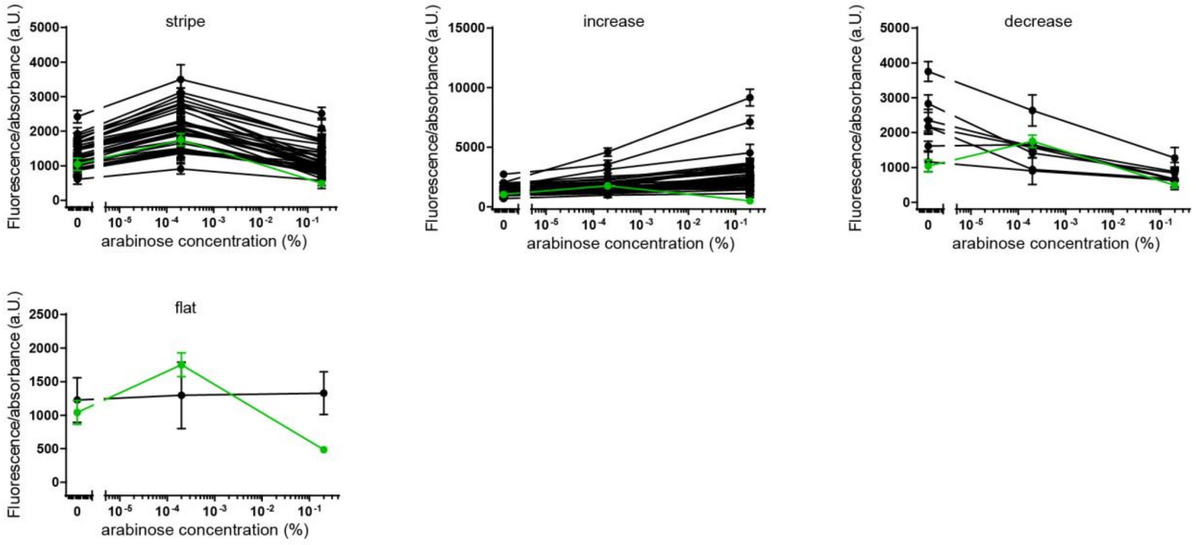

"blue" gene

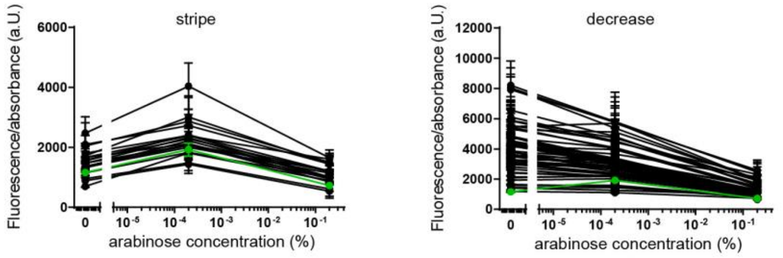

"green" gene

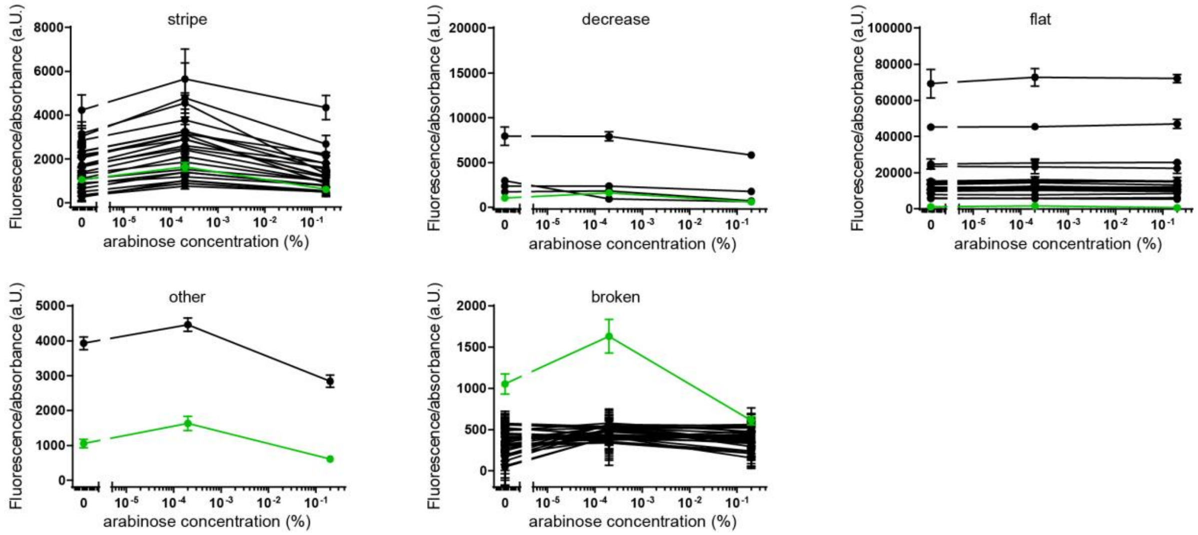

Concurring gradients

"red" gene

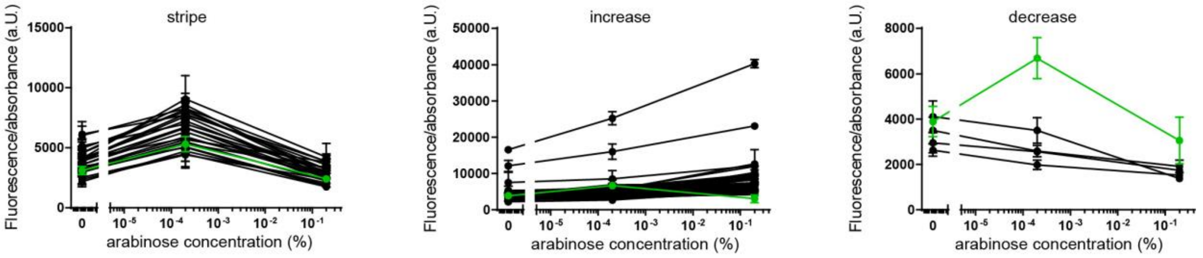

"blue" gene

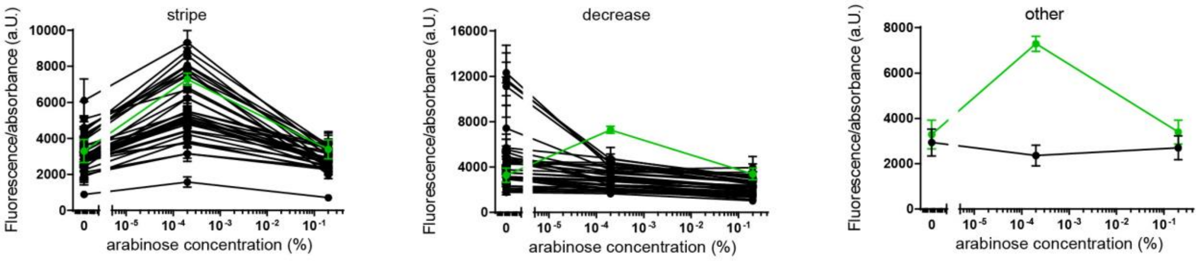

"green" gene

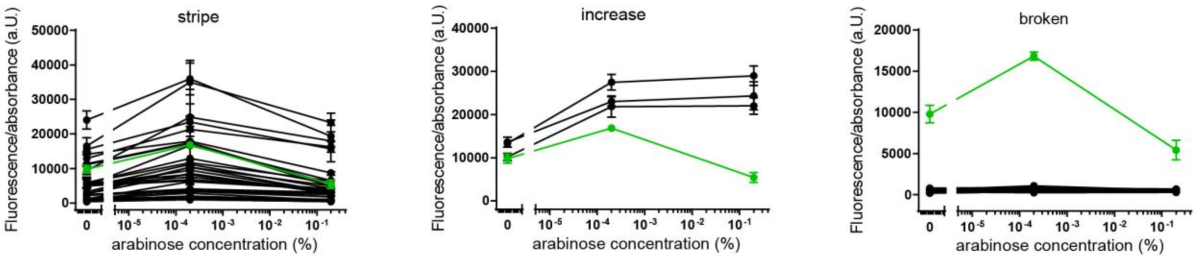

Opposing gradients: mutations in two or three genes

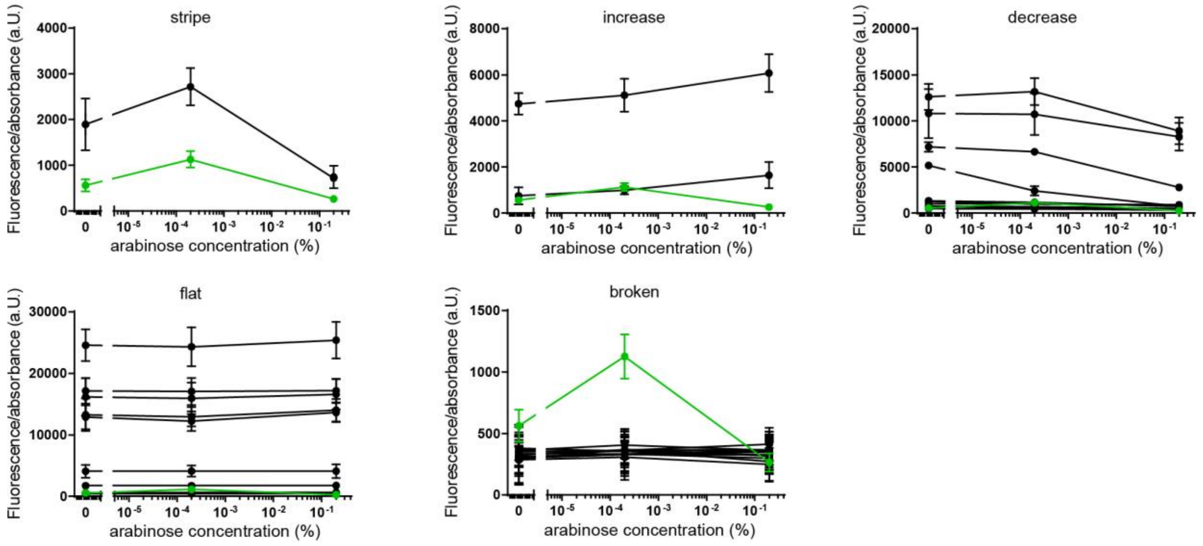

Concurring gradients: mutations in two or three genes

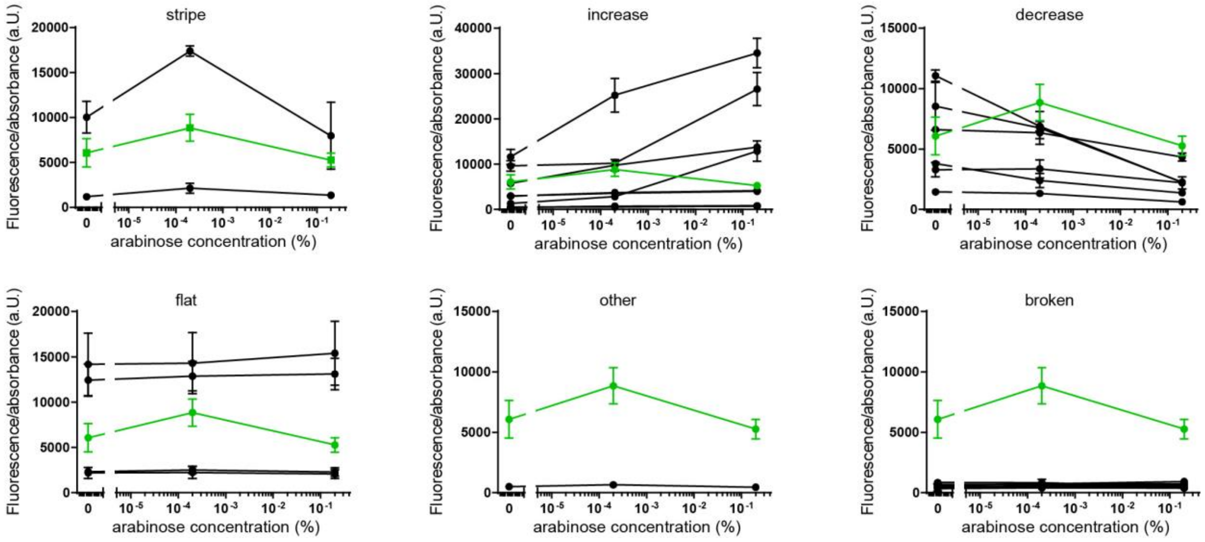

Concurring gradients: mutant A as starting point

“green” gene

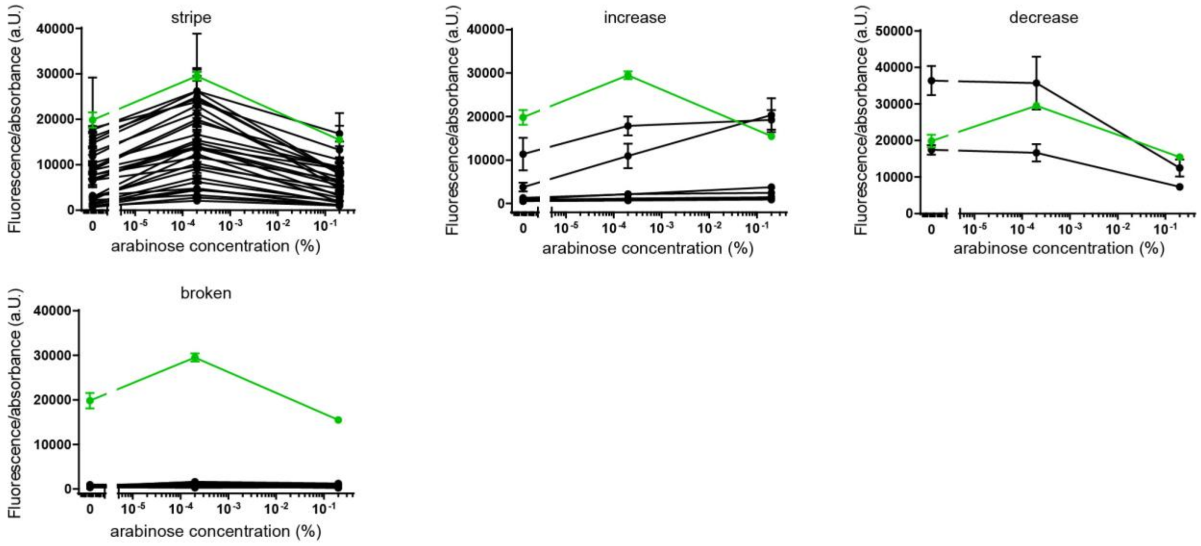

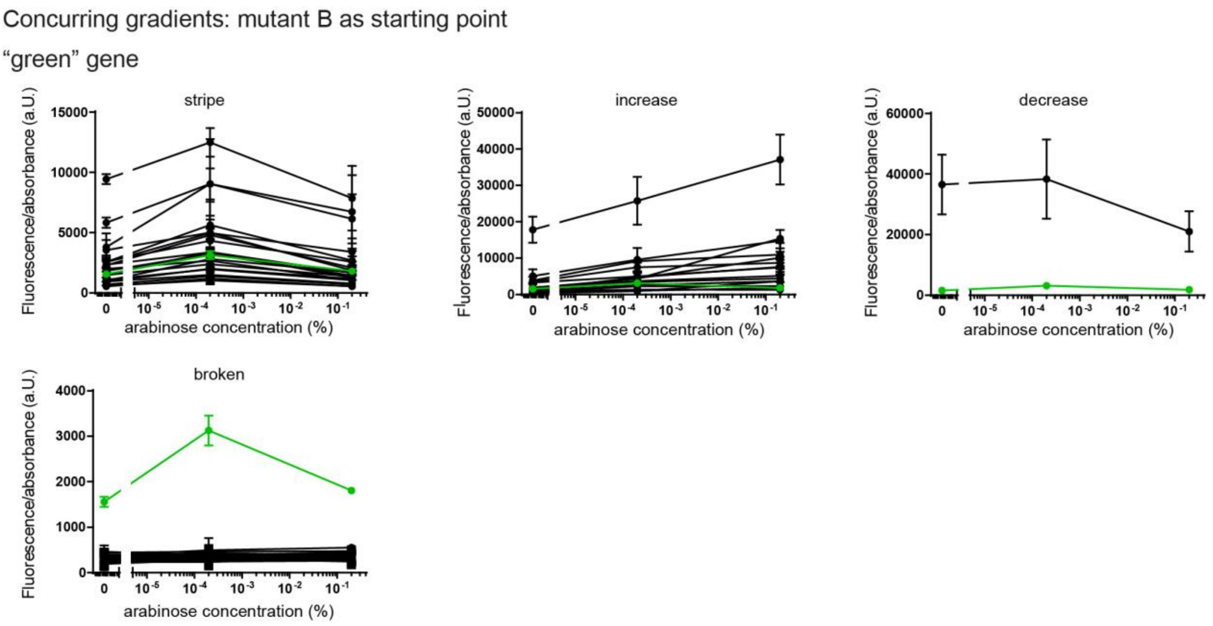

Appendix Figure S1: Phenotypes measured of all mutants. Average and standard deviations of three independent measurements. The green data points belong to the starting network. Lines between the points are for visual guidance only.

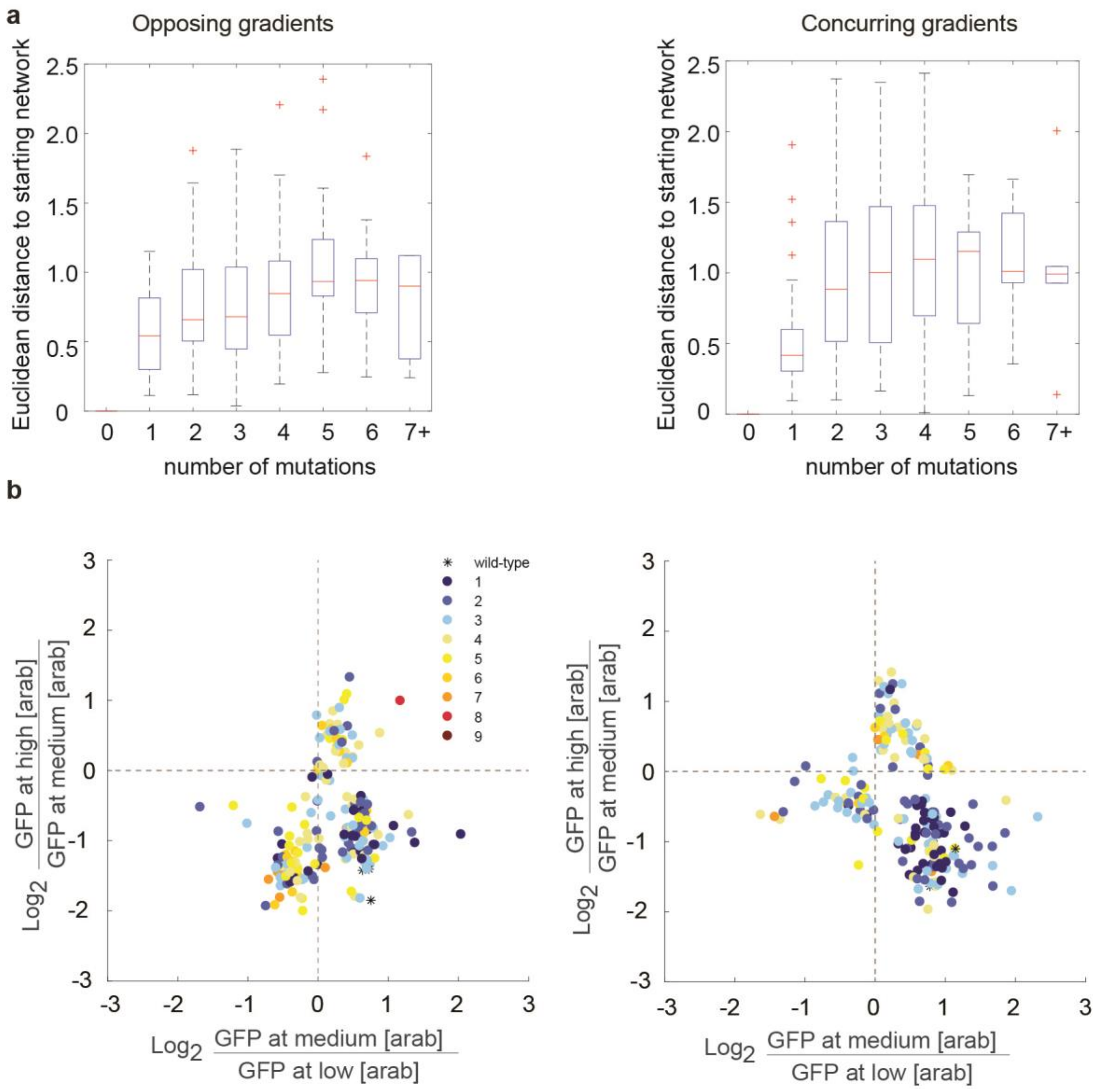

Appendix Figure S2: Correlation between number of mutations and patterns. a Boxplots showing the number of mutations in a network versus their Euclidean distance to the starting wild-type network. The Euclidean distance is calculated by comparing the expression levels of the mutated and wild-type networks at each arabinose concentration. On each box, the central mark indicates the median (red), and the bottom and top edges of the box indicate the 25th and 75th percentiles, respectively. Outliers (data which is more than 1.5 times the interquartile range away from the top or bottom of the box) are plotted using the '+' symbol. There is a weak correlation between the number of mutations and the Euclidean distance (Spearman's correlation = 0.29 for both networks). **b** Relation between number of mutations of a network and distance in pattern space. As shown in Figure 2b, phenotypes cluster and can be separated following the quadrants (vertical and horizontal lines at the origin). Each mutant is coloured according the number of mutations it holds. Mutants with low number of mutations (shades of blue) tend to be located closer to the original stripe networks.

Opposing gradients

"red" gene

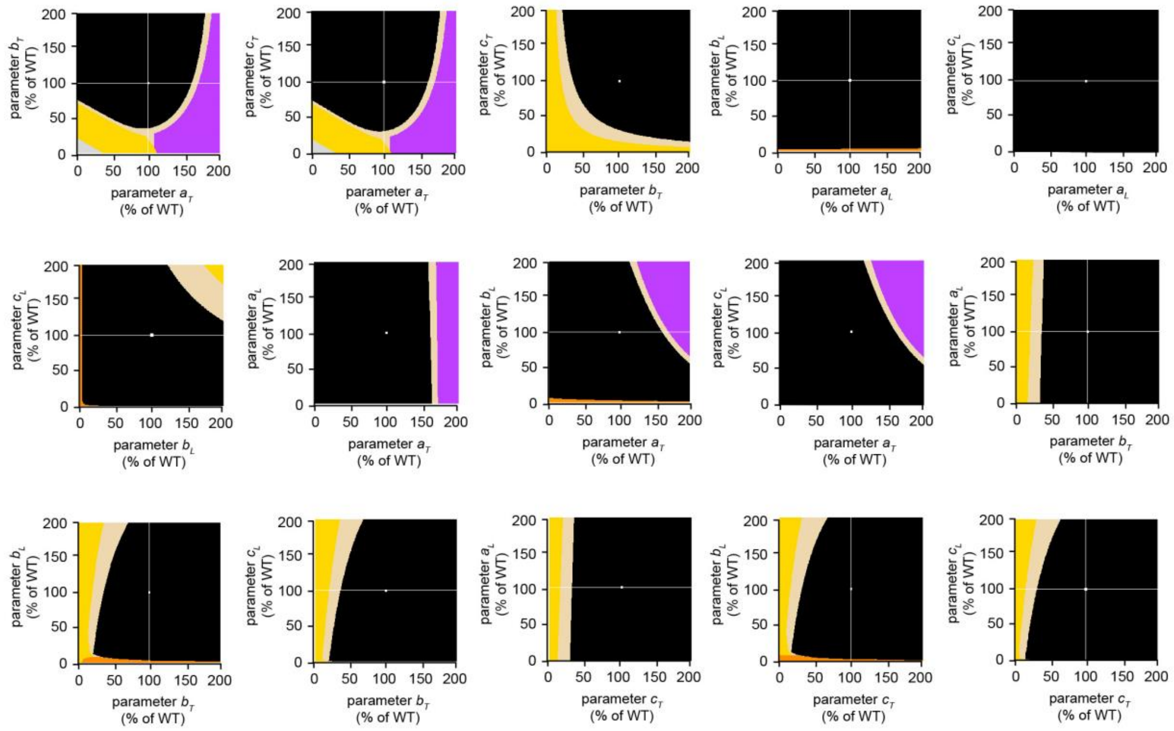

"blue" gene

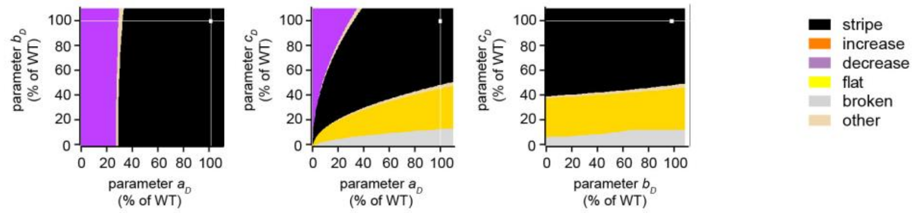

"green" gene

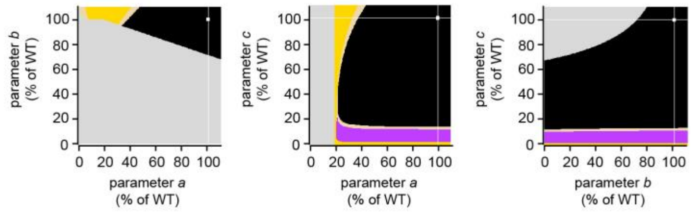

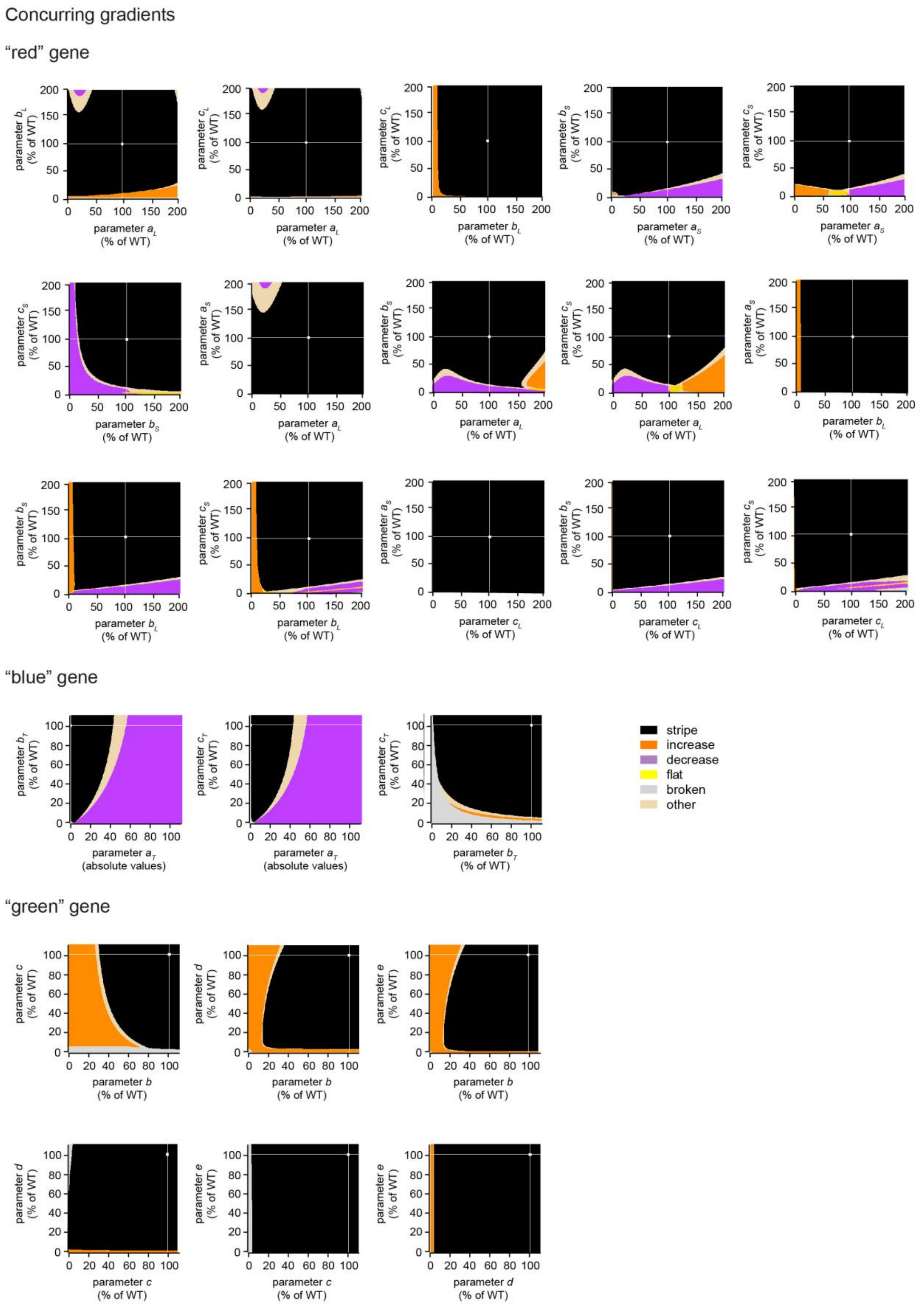

Appendix Figure S3: Phenotype diagrams. Each pair of axes in each panel corresponds to two model parameters and their strength relative to the value in a simulated wild-type circuit.

Each coordinate corresponding to two specific parameter values is assigned a colour corresponding to the model's phenotype at this parameter value. White points represent the parameter combination of the starting WT networks, and white lines represent 100% of the wild-type parameter values. Definitions of the parameters are given in Appendix Tables S1 and S2. The scripts used to make the figures are provided as Computer code EV2.

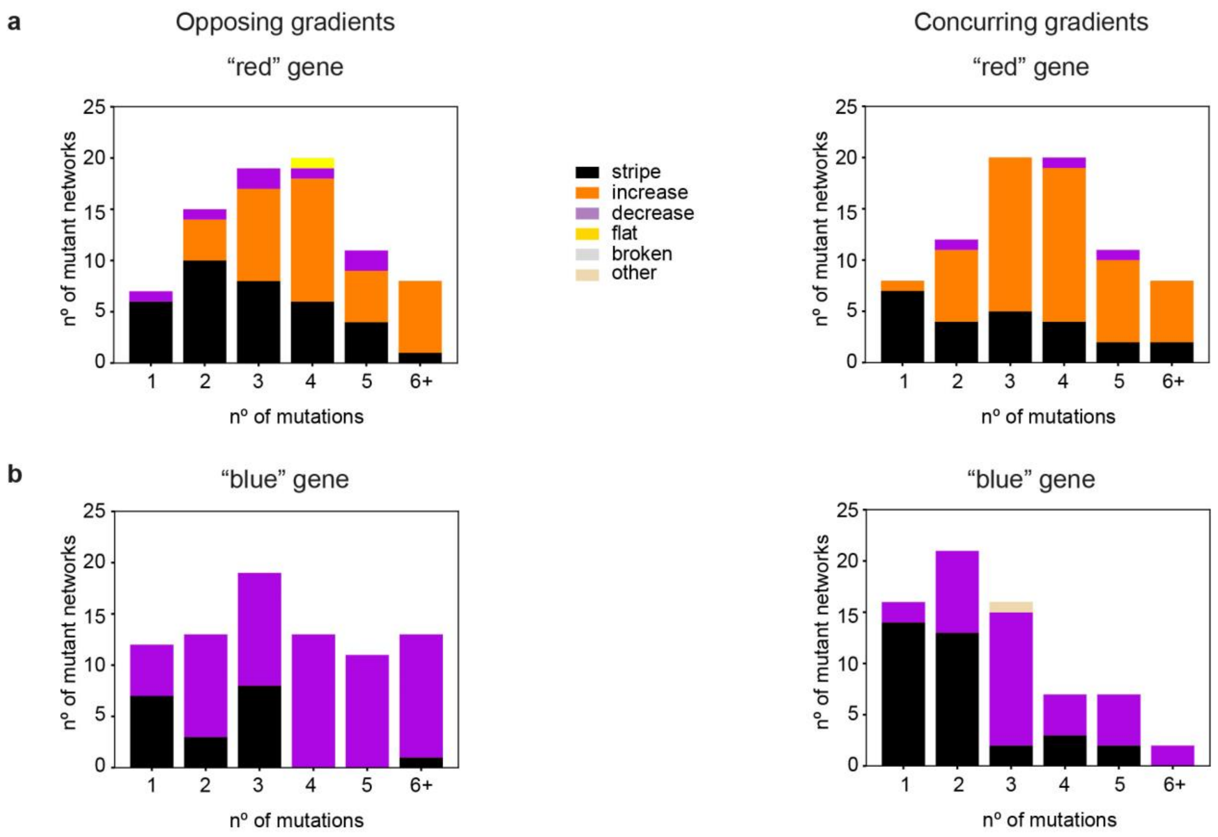

Appendix Figure S4: Number of mutations for the "red" and "blue" genes. Distribution of observed phenotypes of mutants with mutations in the "red" (a) and "blue" (b) gene. Phenotypes are colour-coded (legend).

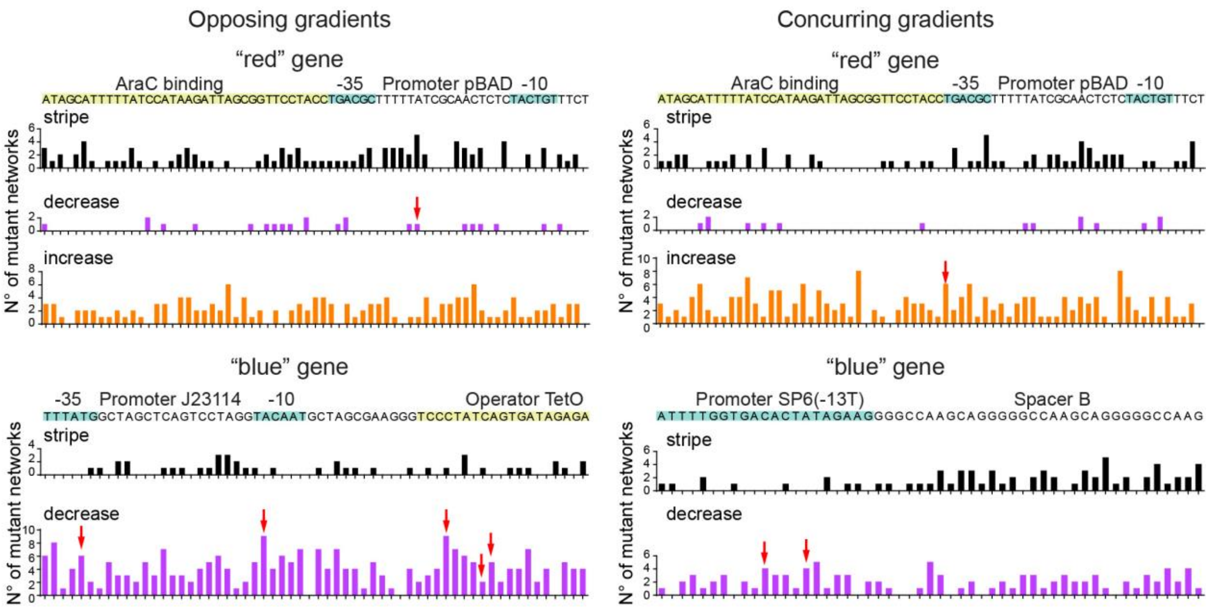

Appendix Figure S5: Summary of all mutations for the "red" and "blue" genes. Wild-type sequences of regulatory regions (top of each panel, important elements labelled and coloured) together with the number of mutations at each site of a regulatory region that produce phenotypes of a given kind (bar-charts below sequence, phenotypes labelled and color-coded). The height of each bar corresponds to the number of mutant networks with a given a mutation at a given position, where these mutations produced the indicated phenotype. Only phenotypes produced by at least three mutant circuits are shown. Red arrows indicate genotypes that can produce a novel phenotype with a single mutation at the indicated position.

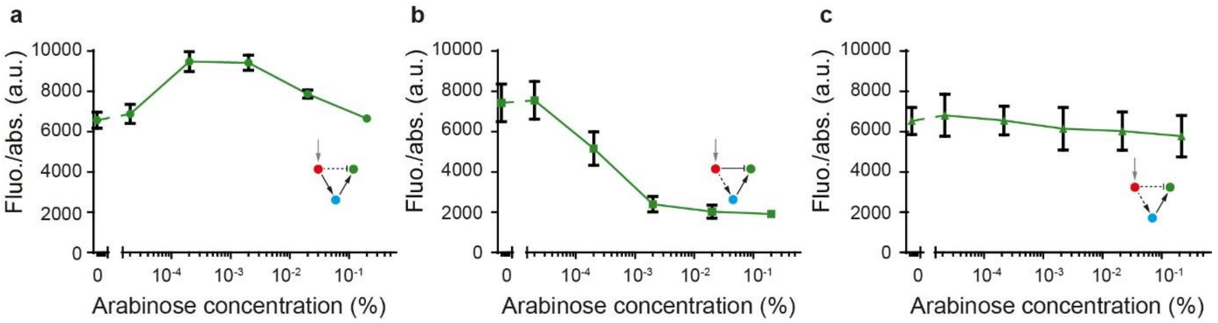

Appendix Figure S6: An experimental example of how non-additive interactions of mutations in multiple regulatory regions in the concurring gradients network produce a flat phenotype. a The "green" gene contains a mutation in the operator, but the network maintains the "stripe" phenotype. **b** A mutation in the "blue" gene promoter leads to a "decrease" phenotype. **c** The combination of the described mutations in the "green" and "blue" gene leads to a "flat" phenotype. Importantly, this new phenotype cannot just be explained as an additive superposition of the two individual phenotypes. Measured fluorescence of the "green" gene (normalised by the absorbance). Mean and s.d. from three biological replicates. Lines between points are for visual guidance only. Insets: Topologies of the concurring gradient network with dashed lines indicating interactions affected by the mutations described.

Appendix Model description

A previously developed and experimentally validated model was used to describe the regulatory dynamics of our networks (Schaerli et al, 2014). The ordinary differential equation systems describing the temporal evolution of the protein concentrations are as follows:

Opposing gradients network

$$\frac{dTetR}{dt} = \frac{\tilde{a}_T + \tilde{b}_T\,(c_T\,ara)^{n_T}}{1 + (c_T\,ara)^{n_T}} - \delta_T TetR \tag{1}$$

$$\frac{dLacI_{inc}}{dt} = \frac{\tilde{a}_L + \tilde{b}_L\,(c_L\,ara)^{n_L}}{1 + (c_L\,ara)^{n_L}} - \delta_L LacI_{inc} \tag{2}$$

$$\frac{dLacI_{dec}}{dt} = \frac{\tilde{a}_D + \tilde{b}_D\,(c_D\,TetR)^{n_L}}{1 + (c_D\,TetR)^{n_L}} - \delta_L LacI_{dec} \tag{3}$$

$$\frac{dLacI}{dt} = \frac{dLacI_{dec}}{dt} + \frac{dLacI_{inc}}{dt} \tag{4}$$

$$\frac{dGFP}{dt} = \frac{\tilde{a} + \tilde{b}\,(c\,LacI)^{n}}{1 + (c\,LacI)^{n}} - \delta GFP \tag{5}$$

Concurring gradients network

$$\frac{dLacI}{dt} = \frac{\tilde{a}_L + \tilde{b}_L\,(c_L\,ara)^{n_L}}{1 + (c_L\,ara)^{n_L}} - \delta_L LacI \tag{6}$$

$$\frac{dSP6}{dt} = \frac{\tilde{a}_S + \tilde{b}_S\,(c_S\,ara)^{n_S}}{1 + (c_S\,ara)^{n_S}} - \delta_S SP6 \tag{7}$$

$$\frac{dT7}{dt} = \frac{\tilde{a}_T + \tilde{b}_T\,(c_T\,SP6)^{n_T}}{1 + (c_T\,SP6)^{n_T}} - \delta_T T7 \tag{8}$$

$$\frac{dGFP}{dt} = \frac{\tilde{a} + \tilde{b}(c\,T7)^{n} + \tilde{e}f(c\,T7)^{n}(d\,LacI)^{m}}{1 + (c\,T7)^{n} + (d\,LacI)^{m} + f(c\,T7)^{n}(d\,lacI)^{m}} - \delta GFP \tag{9}$$

Fig. 1c is reproduced from our initial publication (Schaerli et al, 2014). They are schematic drawings, because we think they are more explanatory than the real simulations which are shown in Appendix Figure S7.

At steady state, the values of the concentrations are given by the equations in Appendix Table S1 and S2, where $\frac{\tilde{a}_T}{\delta_T} = a_T$ (and similarly for the other parameters). Except for Fig. 1c we used the steady state model throughout the entire study.

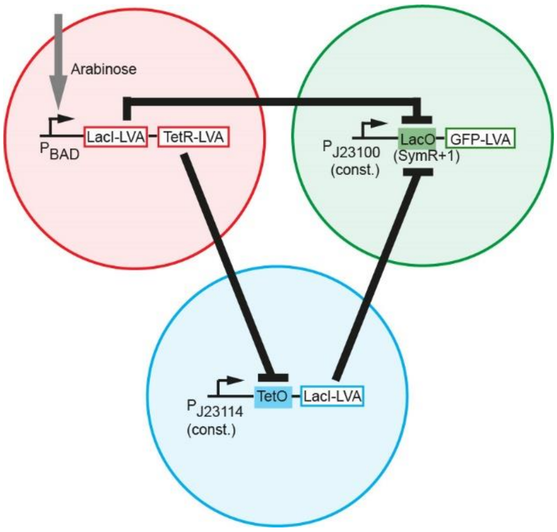

Gene	Name	Value	Definition	Parameter relates to
red	a_T<br>b_T<br>c_T<br>n_T	1.87e+00<br>4.31e+01<br>3.88e+02<br>1	$TetR = \frac{a_T + b_T(c_T\ ara)^{n_T}}{1 + (c_T\ ara)^{n_T}}$	basal transcription rate in absence of arabinose<br>transcription rate when arabinose/AraC is bound<br>binding constant of arabinose/AraC<br>Hill coefficient (multimerization or cooperativity)
red	a_L<br>b_L<br>c_L<br>n_L	5.16e-01<br>1.08e+02<br>1.30e+02<br>1	$LacI_{inc} = \frac{a_L + b_L(c_L\ ara)^{n_L}}{1 + (c_L\ ara)^{n_L}}$	basal transcription rate in absence of arabinose<br>transcription rate when arabinose/AraC is bound<br>binding constant of arabinose/AraC<br>Hill coefficient (multimerization or cooperativity)
blue	a_D<br>b_D<br>c_D<br>n_D	1.24e+04<br>3.98e+00<br>1.65e+01<br>2	$LacI_{dec} = \frac{a_D + b_D(c_D\ TetR)^{n_D}}{1 + (c_D\ TetR)^{n_D}}$	basal transcription rates from the free promoter<br>transcription rate when TetR is bound<br>binding constant of TetR<br>Hill coefficient (multimerization or cooperativity)
green	a<br>b<br>c<br>n	4.40e+02<br>8.47e+01<br>2.52e-01<br>3.22e+00	$LacI = LacI_{inc} + LacI_{dec}$<br><br>$GFP = \frac{a + b(c\ LacI)^n}{1 + (c\ LacI)^n}$	basal transcription rates from the free promoter<br>transcription rate when LacI is bound<br>binding constant of LacI<br>Hill coefficient (multimerization or cooperativity)

Appendix Table S1: Steady state model and fitted parameter values of the opposing gradients network from (Schaerli et al, 2014). This network was previously named incoherent feedforward loop type 2 (I2) (Mangan & Alon, 2003).

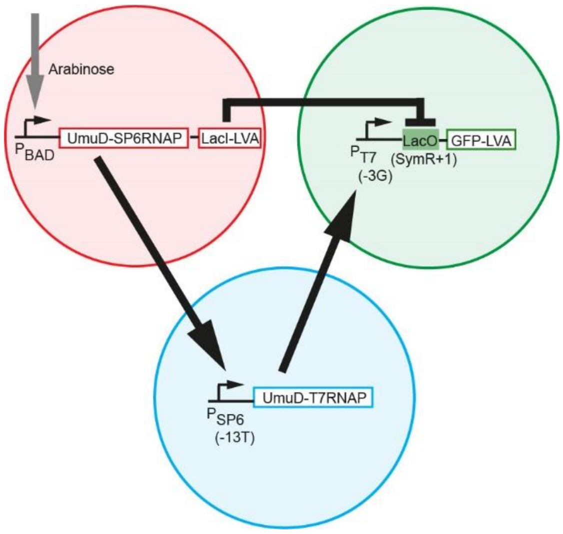

Gene	Name	Value	Definition	Parameter relates to
red	a_L<br>b_L<br>c_L<br>n_L	2.62e+01<br>4.86e+02<br>5.88e+01<br>1	$LacI = \frac{a_L + b_L(c_L\, ara)^{n_L}}{1 + (c_L\, ara)^{n_L}}$	basal transcription rate in absence of arabinose<br>transcription rate when arabinose/AraC is bound<br>binding constant of arabinose/AraC<br>Hill coefficient (multimerization or cooperativity)
red	a_s<br>b_s<br>c_s<br>n_s	7.02e+00<br>3.80e+02<br>8.45e+02<br>1.1	$SP6 = \frac{a_s + b_s(c_s\, ara)^{n_s}}{1 + (c_s\, ara)^{n_s}}$	basal transcription rate in absence of arabinose<br>transcription rate when arabinose/AraC is bound<br>binding constant of arabinose/AraC<br>Hill coefficient (multimerization or cooperativity)
blue	a_T<br>b_T<br>c_T<br>n_T	0<br>1.55e+03<br>1.71e-03<br>1	$T7 = \frac{a_T + b_T(c_T\, SP6)^{n_T}}{1 + (c_T\, SP6)^{n_T}}$	basal transcription rate in absence of SP6 RNAP<br>transcription rate when SP6 RNAP is bound<br>binding constant of SP6 RNAP<br>Hill coefficient (multimerization or cooperativity)
green	a<br>b<br>c<br>d<br>e<br>f<br>n<br>m	0<br>4.43e+03<br>8.76e-02<br>1.03e-01<br>5.42e+02<br>9.84e-02<br>1<br>2.35	$GFP = \frac{a + b(c\,T7)^n + ef(c\,T7)^n(d\,LacI)^m}{1 + (c\,T7)^n + (d\,LacI)^m + f(c\,T7)^n(d\,LacI)^m}$	basal transcription rate in absence of T7 RNAP<br>transcription rate when T7 RNAP is bound<br>binding constant of T7 RNAP<br>binding constant of LacI<br>transcription rate when T7 RNAP + LacI are bound<br>cooperativity/competition constant of T7RNAP/LacI<br>Hill coefficient (multimerization or cooperativity)<br>Hill coefficient (multimerization or cooperativity)

Appendix Table S2: Steady state model and fitted parameter values of the concurring gradients network from (Schaerli et al, 2014). This network was previously named incoherent feedforward loop type 3 (I3) (Mangan & Alon, 2003).

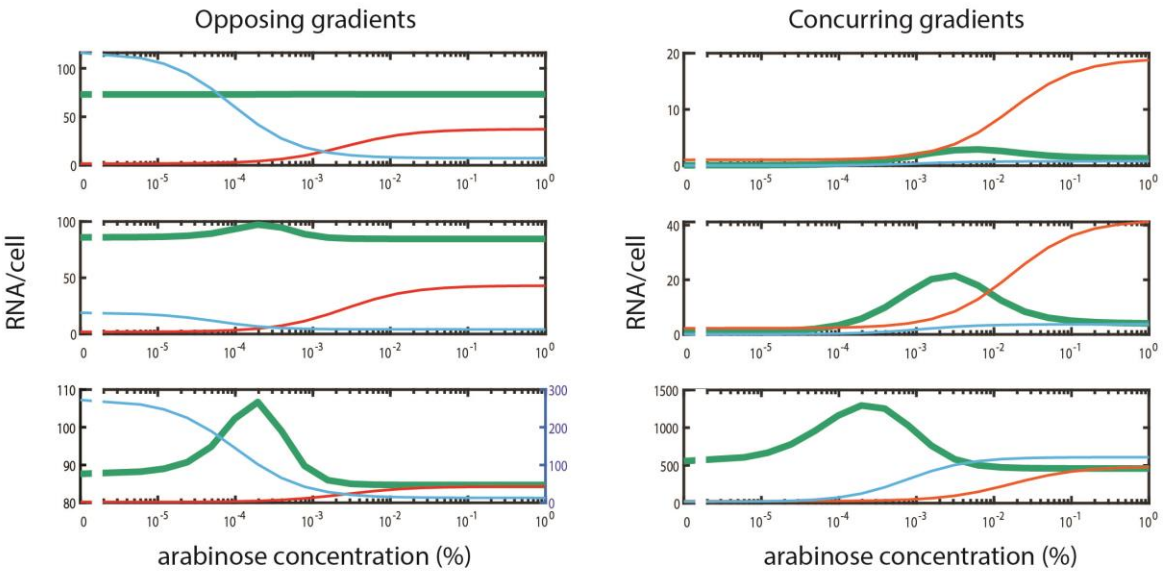

Appendix Figure S7: Simulation of spatiotemporal course of gene expression (colour-code as in Fig. 1) at an early (top), intermediate (middle) and late (bottom) time point (steady state) for the opposing gradients (left) and the concurring gradients (right) networks. Parameters were taken from Appendix Tables S1 and S2. The scripts used to make the figures are provided as Computer code EV1.

Gene	Parameter	Range (% of wildtype value) Lower bound	Upper bound	Comment	Changed when mutation in promoter	Changed when mutation in operator
**Opposing gradients network**						
red	a_T	100	160	varies jointly with a_L	X	X
	b_T	0.1	100		X	X
	c_T	20	100	varies jointly with c_L		X
	a_L	100	160	varies jointly with a_T	X	X
	b_L	0.1	20		X	X
	c_L	20	100	varies jointly with c_T		X
blue	a_D	1	30	varies jointly with b_D	X	
	b_D	1	30	varies jointly with a_D	X	
	c_D	70	100			X
green	a	15	30		X	
	b	95	100		X	
	c	0.1	100			X
**Concurring gradients network**						
red	a_L	100	160	varies jointly with a_S	X	X
	b_L	0.1	20		X	X
	c_L	20	100	varies jointly with c_S		X
	a_S	100	160	varies jointly with a_L	X	X
	b_S	0.1	100		X	X
	c_S	20	100	varies jointly with c_L		X
blue	a_T	1	40	no range, but raw values (the initial value was set to 0)	X	n/a
	b_T	0.1	100		X	n/a
	c_T	0.1	20		X	n/a
green	b	1	80	varies jointly with e	X	
	c	1	9		X	
	d	1	100			X
	e	1	80	varies jointly with b	X	

Appendix Table S3: Lower and upper bounds of intervals (3rd and 4th column from left) for uniform distributions used for simulated mutations of parameters (2nd column from left) to predict the distributions of phenotypes quantitatively. Hill coefficients of both networks and parameters *a* and *f* of the concurring gradients network were not changed. Some parameters were varied jointly and to the same extent (i.e. all of them were changed to same percentage of their wild-type parameter value) (5th column), because a mutation is likely to affect these parameters in a similar way.

Gene	Promoter length (nucleotides)	Operator length (nucleotides)	Single-gene mutants average mutation rate	Multiple-gene mutants average mutation rate
**opposing gradients network**				
red	34	35	3.51	3.03
blue	40	19	3.51	3.3
green	19	26	3.49	2.89
**concurring gradients network**				
red	34	35	3.53	2.61
blue	53	n/a	2.68	2.56
green	19	26	2.62	2.66

Appendix Table S4: Lengths of the regulatory sequence elements and average mutation rates extracted from the experimental data.

	Opposing gradients		Concurring gradients	
	e	m	e	m
stripe	2.8	5.5	4.9	8.0
increase	5.6	2.3	22.0	27.3
decrease	27.8	36.9	14.6	8.5
flat	27.8	23.2	9.8	4.4
broken	36.1	25.9	46.3	48.6
other	0.0	6.2	2.4	3.3

Appendix Table S5: Mutations in multiple regulatory regions interact non-additively. Data underlying Figures 6b/c. e: Experimentally observed phenotype distributions when mutating multiple regulatory regions. m: Phenotype distributions produced by the model when mutating multiple regulatory regions.

Appendix Discussion of lower and upper bounds of the parameter intervals:

The intervals of parameters describing the basal transcription promoter activity (“leakiness”) of the pBAD promoter in both networks (opposing gradients: a_T, a_L, concurring gradients: a_L, a_S) have upper bounds higher than 100% of the unmutated (WT) value (Appendix Table S3). This can be explained by the pBAD promoter DNA looping mechanism (Lobell & Schleif, 1990): In the absence of arabinose AraC represses the pBAD promoter by DNA looping (Lobell & Schleif, 1990). Therefore, mutations that decrease AraC binding in the araI1 region increase the leakiness of the promoter (Lagator et al, 2016; Martin et al, 1986; Schleif, 2010). Since the regulatory sequence of the “red” genes of the two networks are identical, it was reassuring that the parameter ranges our computational procedure determined are nearly identical for both networks (Appendix Table S3 “red” genes).

The basal transcription promoter activity (“leakiness”) of the SP6 promoter in the concurring gradient network (a_T) also has an upper bound higher than 100% of the unmutated (WT) value (Appendix Table S3). This was unexpected and led us to discover a context dependent effect: For the “blue” gene of the concurring gradients network we observe a high percentage of mutants with a “decrease” phenotype. The sequences of the mutants (Dataset EV1) displaying this “decrease” phenotype all have mutations that strongly reduce the SP6 promoter strength (<5% of WT activity) (Shin et al, 2000). Strongly reducing the promoter activity in the model (i.e. parameter b_T), leads to low levels of T7 RNA polymerase and consequently to a “broken” phenotype and not the “decrease” phenotype that we observed experimentally. The model predicts that the “decrease” phenotype is produced when the basal expression (i.e. in the absence of any SP6 RNA polymerase) of the SP6 promoter is increased (parameter a_T). Indeed, many experimentally observed phenotypes of mutant networks have an increased expression level at 0% arabinose compared to the WT network (Appendix Fig. S1). However, the SP6 promoter should not have any basal expression in *E. coli* cells, because the *E. coli* RNA polymerase does not recognise this promoter. To reflect this fact, the parameter a_T was fixed to 0 during our previous study (Schaerli et al, 2014). We now tested the concurring gradients network without a SP6 promoter and indeed observed a “decrease” and not a “broken” phenotype (not shown). That indicates that transcription (probably by the *E. coli* polymerase) can also start from some other site upstream of the promoter in our plasmid.

Appendix References

Lagator M, Igler C, Moreno AB, Guet CC, Bollback JP (2016) Epistatic Interactions in the Arabinose Cis-Regulatory Element. *Mol Biol Evol* **33:** 761-769

Lobell RB, Schleif RF (1990) DNA looping and unlooping by AraC protein. *Science* **250:** 528-532

Mangan S, Alon U (2003) Structure and function of the feed-forward loop network motif. *Proc Natl Acad Sci U S A* **100:** 11980-11985

Martin K, Huo L, Schleif RF (1986) The DNA loop model for ara repression: AraC protein occupies the proposed loop sites in vivo and repression-negative mutations lie in these same sites. *Proc Natl Acad Sci U S A* **83:** 3654-3658

Schaerli Y, Munteanu A, Gili M, Cotterell J, Sharpe J, Isalan M (2014) A unified design space of synthetic stripe-forming networks. *Nat Commun* **5:** 4905

Schleif R (2010) AraC protein, regulation of the l-arabinose operon in Escherichia coli, and the light switch mechanism of AraC action. *FEMS Microbiol Rev* **34:** 779-796

Shin I, Kim J, Cantor CR, Kang C (2000) Effects of saturation mutagenesis of the phage SP6 promoter on transcription activity, presented by activity logos. *Proc Natl Acad Sci U S A* **97:** 3890-3895
