# Supplementary figures and images for: Synthetic circuits reveal how mechanisms of gene regulatory networks constrain evolution
Source: Mol Syst Biol. 2018 Sep 10;14(9):e8102. doi: 10.15252/msb.20178102 (PMC6129954; doi:10.15252/msb.20178102)

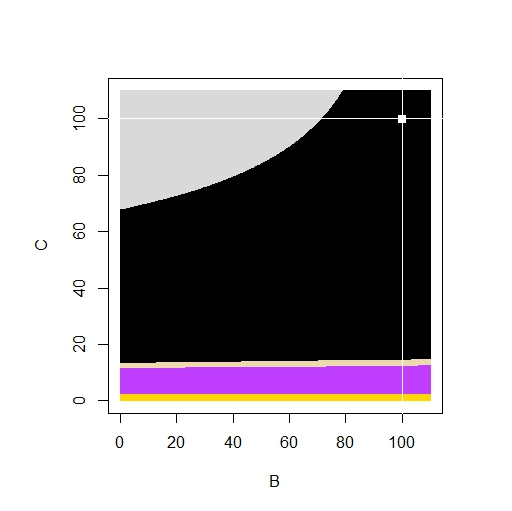

Supplement: Supplementary file 5 — Code EV2 [file MSB-14-e8102-s005.zip › Model_landscapes/I2_c_b.jpeg]

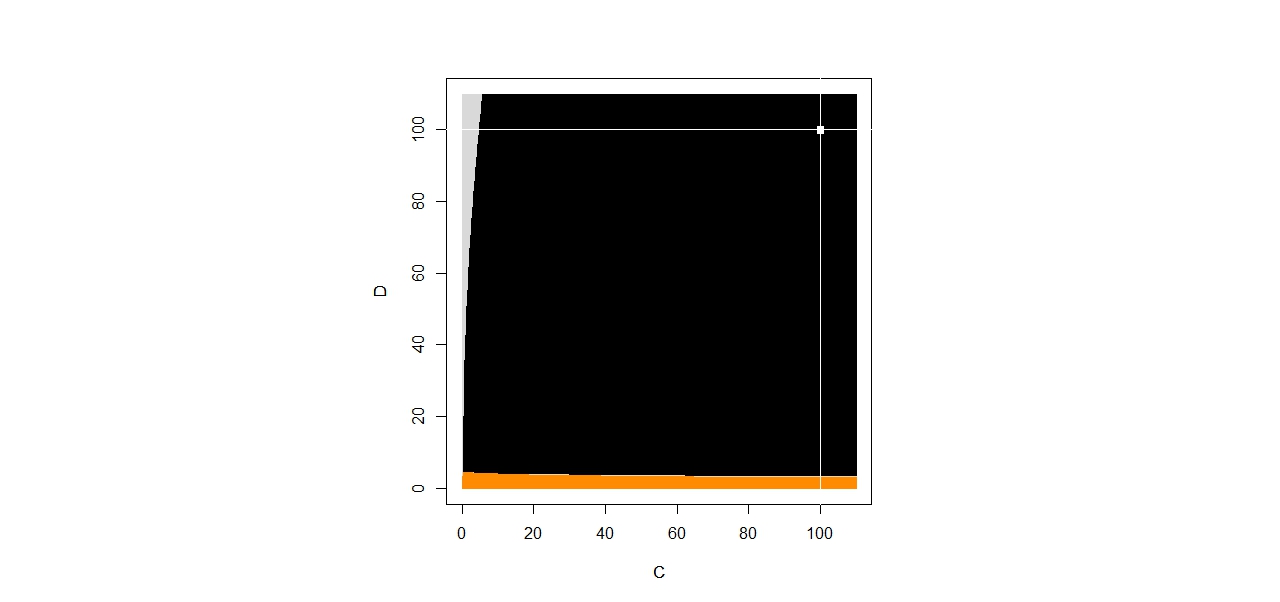

Supplement: Supplementary file 5 — Code EV2 [file MSB-14-e8102-s005.zip › Model_landscapes/I3_d_c.jpeg]

## I2: Green Gene

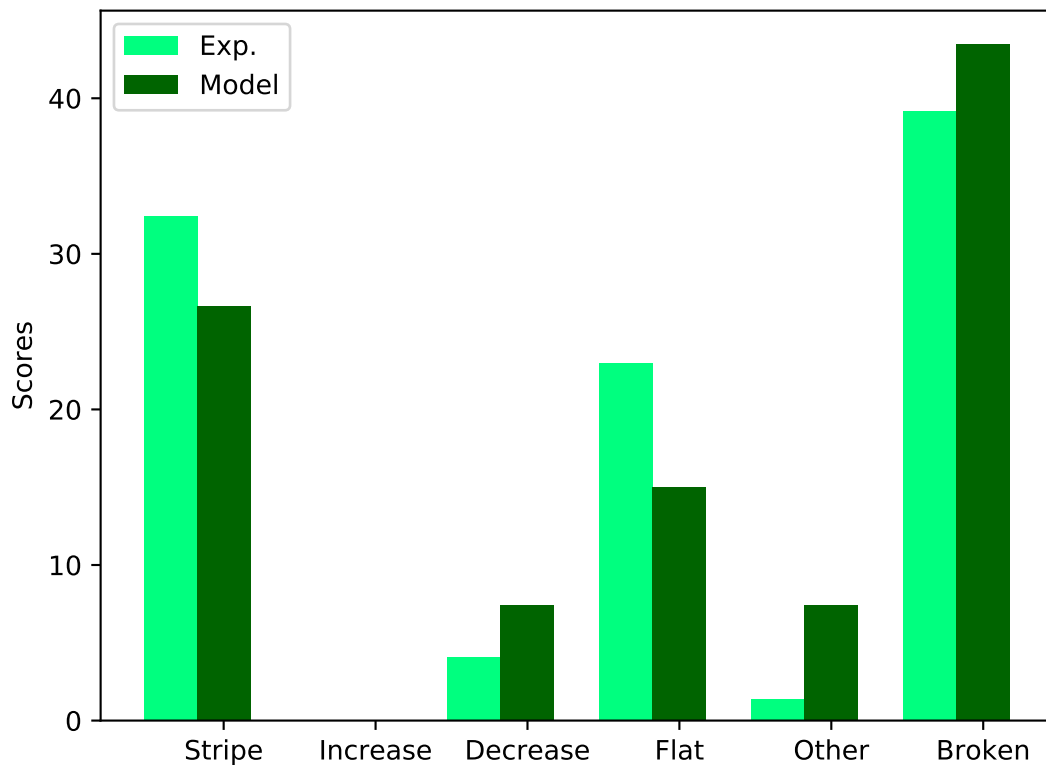

Supplement: Supplementary file 6 — Code EV3 [file MSB-14-e8102-s006.zip › Model_Phenotypic_Distributions/green_I2_model.pdf]

### I3: Green Gene

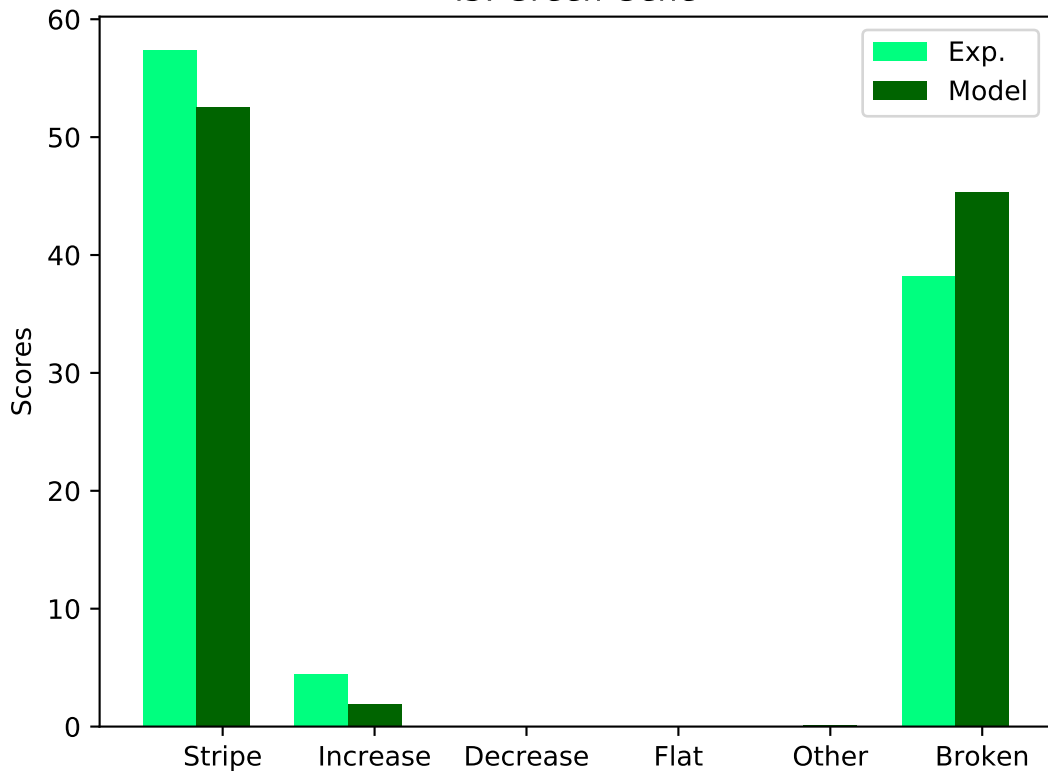

Supplement: Supplementary file 6 — Code EV3 [file MSB-14-e8102-s006.zip › Model_Phenotypic_Distributions/green_I3_model.pdf]
